# Different Effects of Pre-transplantation Measurable Residual Disease on Outcomes According to Transplant Modality in Patients With Philadelphia Chromosome Positive ALL

**DOI:** 10.3389/fonc.2020.00320

**Published:** 2020-03-17

**Authors:** Si-Qi Li, Qiao-Zhen Fan, Lan-Ping Xu, Yu Wang, Xiao-Hui Zhang, Huan Chen, Yu-Hong Chen, Feng-Rong Wang, Wei Han, Yu-Qian Sun, Chen-Hua Yan, Fei-Fei Tang, Yan-Rong Liu, Xiao-Dong Mo, Xin-Yu Wang, Kai-Yan Liu, Xiao-Jun Huang, Ying-Jun Chang

**Affiliations:** ^1^Beijing Key Laboratory of Hematopoietic Stem Cell Transplantation, National Clinical Research Center for Hematologic Disease, Peking University People's Hospital, Peking University Institute of Hematology, Beijing, China; ^2^Peking-Tsinghua Center for Life Sciences, Beijing, China

**Keywords:** haploidentical allografts, Philadelphia-chromosome positive, acute lymphoblastic leukemia, HLA-matched sibling donor transplantation, measurable residual disease

## Abstract

**Background:** This study compared the effects of pre-transplantation measurable residual disease (pre-MRD) on outcomes in Philadelphia chromosome (Ph)-positive ALL patients who underwent human leukocyte antigen-matched sibling donor transplantation (MSDT) or who received unmanipulated haploidentical SCT (haplo-SCT).

**Methods:** A retrospective study (*n* = 202) was performed. MRD was detected by RT-PCR and multiparameter flow cytometry.

**Results:** In the total patient group, patients with positive pre-MRD had a higher 4-year cumulative incidence of relapse (CIR) than that in patients with negative pre-MRD (26.1% vs. 12.1%, *P* = 0.009); however, the cumulative incidence of non-relapse mortality (NRM) (7.4% vs. 15.9%, *P* = 0.148), probability of leukemia-free survival (LFS) (66.3% vs. 71.4%, *P* = 0.480), and overall survival (OS) (68.8% vs. 76.5%, *P* = 0.322) were comparable. In the MSDT group, patients with positive pre-MRD had increased 4-year CIR (56.4% vs. 13.8%, *P* < 0.001) and decreased 4-year LFS (35.9% vs. 71.0%, *P* = 0.024) and OS (35.9% vs. 77.6%, *P* = 0.011) compared with those with negative pre-MRD. In haplo-SCT settings, the 4-year CIR (14.8% vs. 10.7%, *P* = 0.297), NRM (7.3% vs. 16.3%, *P* = 0.187) and the 4-year probability of OS (77.7% vs. 72.3%, *P* = 0.804) and LFS (80.5% vs. 75.7%, *P* = 0.660) were comparable between pre-MRD positive and negative groups. In subgroup patients with positive pre-MRD, haplo-SCT had a lower 4-year CIR (14.8% vs. 56.4%, *P* = 0.021) and a higher 4-year LFS (77.7% vs. 35.9%, *P* = 0.036) and OS (80.5% vs. 35.9%, *P* = 0.027) than those of MSDT. Multivariate analysis showed that haplo-SCT was associated with lower CIR (HR, 0.288; *P* = 0.031), superior LFS (HR, 0.283; *P* = 0.019) and OS (HR, 0.252; *P* = 0.013) in cases with a positive pre-MRD subgroup.

**Conclusions:** Our results indicate that the effects of positive pre-MRD on the outcomes of patients with Ph-positive ALL are different according to transplant modality. For Ph-positive cases with positive pre-MRD, haplo-SCT might have strong graft-vs.-leukemia (GVL) effects.

## Introduction

Philadelphia chromosome (Ph) positivity is one of the most unfavorable cytogenetic prognostic factors in acute lymphoblastic leukemia (ALL), comprising 3–5% children (1), 5–15% adolescents (2) and 25–40% adults (2). The probability of 5-year overall survival (OS) of this subgroup of cases is approximately 30–45% (3–7), although the outcomes have been remarkably improved with the combination of tyrosine kinase inhibitors (TKI) and multiagent chemotherapy. Currently, allogeneic stem cell transplantation (allo-SCT) is a curable therapy for patients with Ph-positive ALL. However, hematological relapse remains one of the major causes of death after allo-SCT (8). Thus, prediction and intervention before leukemia hematological relapse are important in reducing the cumulative incidence of relapse (CIR) and improving transplant outcomes.

Many studies suggest that measurable residual disease (MRD) is an independent prognostic factor in ALL patients who were treated with chemotherapy alone or allo-SCT, making detection of MRD a tool to predict relapse and criteria of risk stratification (9–28). Cazzaniga et al. (29) indicated that Ph-positive ALL patients with negative MRD after consolidation had a lower risk of relapse compared to those with positive MRD. Mizuta et al. (20) demonstrated that negative pre-transplantation MRD (pre-MRD) status, as detected by real-time quantitative polymerase chain reactions (RT-PCR), is associated with significantly lower incidences of relapse in Ph-positive ALL patients who underwent allo-SCT in CR1. Similar results were observed by Ruggeri et al. (21) in pediatric patients with ALL who underwent umbilical cord blood transplantation (UCBT). Zhao et al. (30) indicates that in patients with Ph-positive ALL, MRD detected at early stages after allo-SCT is an important predictor of patient outcomes. Nevertheless, these studies mainly focused on human leukocyte antigen (HLA)-matched sibling donor transplantation (MSDT), HLA-matched unrelated donor transplantation (MUDT) and UCBT.

In the past 10 years, the routine use of haploidentical SCT (haplo-SCT) has allowed almost all patients to undergo allo-SCT (31). Our previous study showed that treating ALL patients with haplo-SCT could achieve outcomes comparable to those of MSDT (32). A study on behalf of the Acute Leukemia Working Party of the Europe Bone Marrow Transplantation (EBMT) (33) suggests that unmanipulated haploidentical allografts could be considered an alternative option for adult patients with high-risk ALL who lack HLA-identical donors, preferably in early disease status. Currently, few data concentrate on the effects of pre-transplantation MRD (pre-MRD) on transplant outcomes in patients with ALL who underwent haplo-SCT (34). Therefore, in this study, we aimed to evaluate the effects of pre-MRD determined by MFC on clinical outcomes in patients with Ph-positive ALL who underwent haplo-SCT. We also investigated whether there were differences in the impacts of pre-MRD on outcomes between Ph-positive ALL patients who underwent haplo-SCT and those of patients who received MSDT.

## Patients and Methods

### Study Design

Two hundred and two Ph-positive ALL patients including children (*n* = 36) and adults (*n* = 166) who underwent MSDT (*n* = 61) and haplo-SCT (*n* = 141) were retrospectively enrolled in this study between March 2011 and December 2016. All of the included subjects provided written informed consent. The study was conducted in accordance with the Declaration of Helsinki and was approved by the Institutional Review Board of Peking University.

### Chemotherapy Before Transplantation

The induction chemotherapy regimen included daunorubicin, cyclophosphamide (Cy), vincristine, prednisone (VDCP), and L-asparaginase or Cy, daunorubicin, vindesine, prednisone (CODP). Consolidation chemotherapy regimen included hyper-CVAD (B) (methotrexate and cytosine arabinoside), high-dose methotrexate with/without L-asparaginase, and the VDCP or CODP regimen, which were given in turn. Prophylaxis for central nervous system leukemia was given to every enrolled patient, which consisted of intrathecal chemotherapy with methotrexate, cytosine arabinoside, and dexamethasone for at least four doses during induction and consolidation chemotherapy (35, 36).

### Transplant Protocol

Unmanipulated haplo-SCT and MSDT were performed according to the protocols reported previously by our group (8, 32).

### Tyrosine Kinase Inhibitors (TKI) Treatment Before and After Transplantation

All Ph-positive ALL patients were treated with a TKI, mainly imatinib, as induction and/or consolidation therapy before transplantation (37). A TKI, usually imatinib, was administered depending on the blood cell counts or the molecular level of the BCR-ABL fusion gene 1, 2. Treatment with imatinib was initiated (1) if patient peripheral blood absolute neutrophil counts were >1.0 × 10^9^/L without granulocyte colony-stimulating factor administration, and the platelet count was >50.0 × 10^9^/L, regardless of the level of BCR-ABL transcript; or (2) if the level of BCR-ABL transcript in the bone marrow was detectable and transcript levels increased for two consecutive tests, or if the BCR-ABL transcript level was ≥10^−2^ after the initial engraftment, although patients' absolute neutrophil counts or platelet count were below the above values. Other criteria for initiation of treatment included that patients could tolerate oral imatinib without gut graft-vs.-host disease (GVHD) or life-threatening infection. Imatinib treatment was scheduled for 3–12 months after hematopoietic cell transplantation, until BCR-ABL transcript levels were negative at least for three consecutive tests or complete molecular remission was sustained for at least 3 months. The initial dose of imatinib was 400 mg/day for adults (age > 17 years) and 260 mg/m^2^/day for children (age < 17 years) 1, 2, 16. The daily dose of imatinib was adjusted according to the National Comprehensive Cancer Network practice guidelines regarding the management of imatinib toxicity (2005 version).

### Donor Lymphocyte Infusion (DLI)

The indications for DLI included hematological leukemia relapse, receiving chemotherapy followed by DLI, molecular test results that provided evidence of persistent leukemia or recurrence in subjects without graft-vs.-host disease (GVHD), and graft failure (GF). The DLI protocol was applied according to our previous study (38–40). For relapse treatment, induction chemotherapy followed by DLI and GVHD prophylaxis was given. For relapse prophylaxis or GF, only DLI and GVHD prevention were used.

### MRD Detection

The BCR-ABL transcript levels in the bone marrow of patients were detected through RT-PCR with ABL as the control gene. Five milliliters of fresh bone marrow (BM) was collected. Samples obtained in EDTA were treated within 2 h of collection to lyse the red blood cells. RNA extraction, complementary DNA synthesis, and RT-PCR analysis were performed as previously described in the literature (41). ABL was selected as a control gene to compensate for variations in the quality and quantity of RNA and cDNA. BCR-ABL primers and probes that amplified both b3a2 and b2a2 junctions were designed using Primer Express software version 2.0. There were similar primers and probes described in Europe against Cancer Program report (41). The primer and probe sequences of BCR-ABL mRNA have been described previously (42).

ABL primers and probes were referred to in the report of the Europe Against Cancer Program (43). The copy numbers of all ABL samples were more than 3 × 10^4^. The reproducible sensitivity of PCR was five copies. All experiments were performed in duplicate. If BCR-ABL mRNA was detected, the sample was considered positive, and the number of transcripts was calculated as BCR-ABL/ABL %. If BCR-ABL mRNA was undetected, the sample was regarded as negative, and BCR-ABL/ABL% was equal to zero. The molecular responses in PB and BM samples were defined as the log-reductions of BCR-ABL mRNA level from the baseline value of PB and BM, respectively, which were the median levels from newly diagnosed CP CML patients. Major molecular response (MMR) in PB and BM samples were defined as ≥3 log-reductions of BCR-ABL mRNA level from the baseline value of PB and BM, respectively. MRD negative and MRD positive were defined as not detectable and detectable as previous report by Yanada et al. (44) The threshold for quantification was 50 copies/μg RNA, which corresponded to a sensitivity of 10^−5^.

The MRD was also determined by multiparameter flow cytometry (MFC) according to previous publication (30). A lower limit of detection (LOD) of 0.001% was targeted.

MRD detection was performed in all patients as a routine clinical test on bone marrow aspirate samples that were obtained at 1 month before SCT as well as at days 30, 60, 90, 120, and 180 after transplantation (37).

In this study, positive pre-MRD was defined using a cutoff value of 0.001% determined by MFC according to our publication (34). In our previous study, we showed that for ALL patients who underwent haplo-SCT, cases with positive pre-MRD (≧ 0.001%) detected by MFC had a significantly higher CIR than that of cases with negative one (<0.001%) (34).

### Outcomes

The primary study endpoint was the CIR. The secondary endpoints were the cumulative incidence of non-relapse mortality (NRM), the probability of leukemia-free survival (LFS) and overall survival (OS). Engraftment, infection, NRM, relapse, LFS, OS, acute GVHD, and chronic GVHD were defined as previously described (38, 45, 46).

### Propensity Score Matched Analysis

Propensity score matched analysis was performed by attempting to match each patient who underwent MSDT with those who underwent haplo-SCT (a 1:1 match). Using the nearest-neighbor-matching method, propensity score matching was performed using the following parameters: sex, age, pre-MRD. A match occurred when the difference in logits of propensity score was <0.2 times the standard deviation of scores.

### Statistical Analysis

Patient characteristics were compared between the positive pre-MRD and negative using χ^2^ statistic categorical variables and the Mann-Whitney test for continuous variables. The probability of relapse, non-relapse mortality (NRM), LFS and OS were estimated with the Kaplan-Meier method. NRM was defined as death without relapse and was treated as a competing risk for relapse. However, relapse was considered a competing risk for NRM. MRD status pre-transplantation and all variables in Table 1 were included in the univariate analysis. Only variables with *P* < 0.1 were included in a Cox proportional hazards model with time-dependent variables. Unless otherwise specified, *P*-values were based on two-sided hypothesis tests. Alpha was set at 0.05. Most analyses were performed with SPSS 16.0 (Mathsoft, Seattle, WA, USA).

**Table 1:** Patient and donor characteristics (*n* = 202).

**Characteristic**	**All patients**	**MSDT**			**Haplo-SCT**		
		**MRD neg**	**MRD pos**	***P*-value**	**MRD neg**	**MRD pos**	***P*-value**
Number of patients	202	48	13		100	41	
Median age (range), years	32 (4–63)	40 (7–63)	38 (8–60)	0.828	27 (4–57)	32 (11–53)	0.368
Male, n (%)	117 (57.9%)	23 (47.9%)	6 (46.2%)	0.910	60 (60.0%)	28 (68.3%)	0.356
Disease status, n (%)				0.006			0.003
CR1	188 (93.1%)	47 (97.9%)	9 (69.2%)		98 (98.0%)	34 (82.9%)	
CR2	13 (6.9%)	1 (2.1%)	4 (30.8%)		2 (2.0%)	6 (14.7%)	
CR > 2	1 (0.5%)	0	0		0	1 (2.4%)	
IKZF, n (%)				0.409			0.890
Positive	49 (24.3%)	9 (18.8%)	4 (30.8%)		20 (20.0%)	16 (39.0%)	
Negative	57 (28.2%)	9 (18.8%)	3 (23.1%)		35 (35.0%)	10 (24.4%)	
Unknown	96 (47.5%)	30 (62.5%)	6 (46.2%)		45 (45.0%)	15 (36.6%)	
Cytogenetic subgroup of ALL, n (%)				1.000			0.770
t(9;22)	136 (67.3%)	35 (72.9%)	9 (69.2%)		66 (66.0%)	26 (63.4%)	
t(9;22) with other karyotypic abnormalities	66 (32.7%)	13 (27.1%)	4 (30.8%)		34 (34.0%)	15 (36.6%)	
WBC at diagnosis, n (%)				0.850			0.254
High	98 (48.5%)	16 (33.3%)	5 (38.4%)		53 (53.0%)	24 (58.5%)	
Normal	79 (39.1%)	28 (58.3%)	7 (53.9%)		30 (30.0%)	14 (34.1%)	
Unknown	25 (12.4%)	4 (8.4%)	1 (7.7%)		17 (17.0%)	3 (7.3%)	
Levels of pre–transplant MRD, median (range)	0.03% (0.001–2.210%)		0.03% (0.001–0.760%)			0.03% (0.001–2.210%)	0.670
Donor-recipient sex-matched grafts, n (%)				0.731			0.320
Male-male	47 (23.3%)	12 (25.0%)	2 (15.4%)		20 (20.0%)	13 (31.7%)	
Male-female	35 (17.3%)	13 (27.1%)	5 (38.5%)		13 (13.0%)	4 (9.8%)	
Female-male	71 (35.1%)	12 (25.0%)	4 (30.8%)		40 (40.0%)	15 (36.6%)	
Female-female	49 (24.3%)	11 (22.9%)	2 (15.4%)		27 (27.0%)	9 (22.0%)	
Donor-recipient relationship, n (%)				0.600			0.649
Father-child	47 (23.3%)	0	0		35 (35.0%)	12 (29.3%)	
Mother-child	15 (7.4%)	0	0		10 (10.0%)	5 (12.2%)	
Sibling-sibling	105 (52.0%)	47 (97.9%)	13 (100%)		34 (34.0%)	11 (26.8%)	
Child-parent	30 (14.9%)	0	0		19 (19.0%)	11 (26.8%)	
Other	5 (2.5%)	1 (2.1%)	0		2 (2.0%)	2 (4.9%)	
ABO matched graft, n (%)				0.657			0.153
Matched	117 (57.9%)	31 (64.6%)	10 (76.9%)		57 (57.0%)	19 (46.3%)	
Major mismatch	45 (22.3%)	8 (16.7%)	2 (15.4%)		20 (20.0%)	15 (36.6%)	
Minor mismatch	30 (14.9%)	7 (14.6%)	1 (7.7%)		18 (18.0%)	4 (9.8%)	
Bi-directional mismatch	10 (5.0%)	2 (4.2%)	0		5 (5.0%)	3 (7.3%)	
Cell compositions in allografts							
Infused nuclear cells, (range) 10^8^/kg	8.02 (2.53–13.14)	7.57 (2.53–13.14)	7.99 (5.75–11.41)	0.467	8.22 (4.92–12.06)	8.12 (5.89–11.94)	0.404
Infused CD34^+^ cells, (range) 10^6^/kg	2.82 (0.59–8.51)	2.77 (0.59–8.51)	2.54 (0.59–8.51)	0.664	2.82 (0.62–6.67)	2.96 (0.84–7.20)	0.108
DLI after transplant, n (%)				1.000			0.205
For relapse prophylaxis and intervention	7 (31.8%)	1 (20.0%)	2 (40.0%)		4 (57.1%)	0	
For relapse treatment	15 (68.2%)	4 (80.0%)	3 (60.0%)		3 (42.9%)	5 (100%)	
Pre-transplantation TKI				0.012			0.138
Imatinib	163 (80.7%)	44 (91.7%)	8 (61.5%)		82 (82.0%)	29 (70.7%)	
Others	39 (19.3%)	4 (8.3%)	5 (38.5%)		18 (18.0%)	12 (29.3%)	
Post-transplantation TKI				0.004			0.069
Imatinib	178 (88.1%)	44 (91.7%)	7 (53.8%)		93 (93.0%)	34 (82.9%)	
Others	24 (11.9%)	4 (8.3%)	6 (46.2%)		7 (7.0%)	7 (17.1%)	
Median courses of chemotherapy	4 (1–16)	3 (2–9)	5 (2–16)	0.055	4 (1–11)	4 (2–15)	0.480

*HLA, human leukocyte antigen; MSDT, vHLA-matched sibling donor transplantation; Haplo-SCT, unmanipulated haploidentical stem cell transplantation; MRD, minimal residual disease; neg, negative, pos, positive; CR, complete remission; DLI, donor lymphocyte infusions; TKI, tyrosine kinase inhibitor*.

## Results

### Patient Characteristics

All 202 patients had <5% bone marrow blasts and met the morphological criteria for a leukemia-free state and CR. The median time from diagnosis to SCT was 6 months (2.5–25.0 months). All patients (*n* = 202) achieved sustained, full-donor chimerism and stable neutrophil engraftment. The characteristics of these patients are summarized in Table 1. Both in the MSDT group and the haplo-SCT group, the percentages of cases with ≥CR2 were significantly higher in patients with positive pre-MRD than those of subjects with negative MRD (*P* < 0.05 for all, Table 1). The cumulative incidence of grade II–IV acute GVHD was 21.5%. After a median follow-up of 1,001 days (range, 24–2,575 days), the 4-year cumulative incidence of chronic GVHD was 47.7%. The 4-year CIR and TRM were 15.7 and 13.7%, respectively. The 4-year LFS and OS were 70.2 and 74.5%, respectively.

### Correlation of Pre-MRD With Outcomes in Total Cases who Received allo-SCT

**Figure 1:**
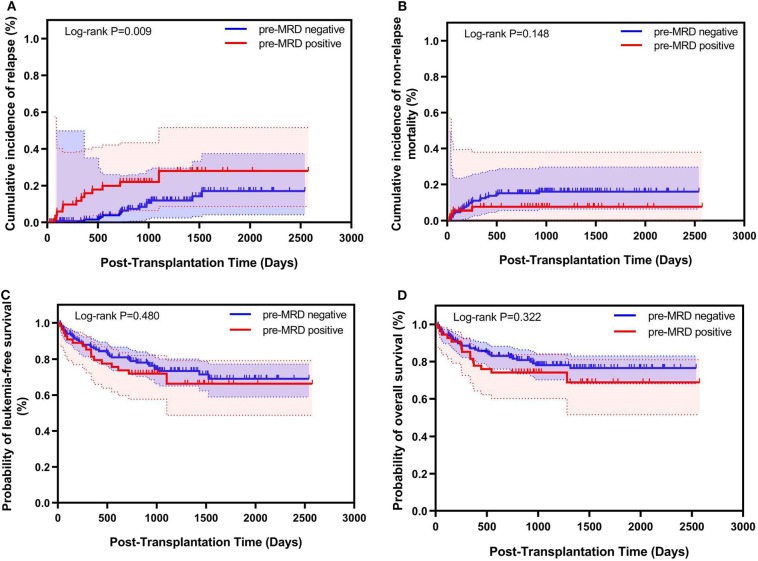
Relationship between pre-transplantation MRD and transplant outcomes for Ph-positive ALL patients who underwent allo-SCT (*n* = 202). Kaplan–Meier estimates of **(A)** cumulative incidence of relapse mortality, **(B)** cumulative incidence of non-relapse, **(C)** leukemia-free survival, and **(D)** overall survival.

Among all 202 Ph-positive ALL patients, 54 (26.7%) had positive pre-MRD. Kaplan-Meier analysis showed that patients with positive pre-MRD had a higher 4-year CIR (26.1% vs. 12.1%, *P* = 0.009) compared to those with negative pre-MRD. The 4-year NRM, OS and LFS were comparable between patients with positive pre-MRD and those with negative pre-MRD (NRM 7.4% vs. 15.9%, *P* = 0.148; OS 68.8% vs. 76.5%, *P* = 0.322; LFS 66.3% vs. 71.4%, *P* = 0.480) (Figure 1). Multivariate analysis showed an association of disease status with CIR (HR, 4.079; *P* = 0.001) (Table 2).

**Table 2:** Multivariate analysis of factors associated with outcomes of patients with Ph positive ALL who underwent allo-SCT (*n* = 202).

**Covariate**	**Univariate analysis**			**Multivariate analysis**		
	**HR**	**95% CI**	***P*-value**	**HR**	**95% CI**	***P*-value**
**Relapse**						
Disease status^*^	2.953	1.500–5.815	0.002	4.079	1.821–9.137	0.001
Pre-transplantation MRD (positive vs. negative)	2.584	1.206–5.539	0.015			
Donor-recipient sex-matched graft						
Female-male	0.928	0.232–3.710	0.916	0.518	0.112–2.392	0.399
Female-female	3.817	1.196–12.181	0.024	3.248	1.006–10.484	0.049
Male-male	1.655	0.509–5.378	0.402	1.464	0.448–4.789	0.528
Male-female		1			1	
**Transplant–related mortality**						
Platelet engraftment (yes vs. no)	0.047	0.020–0.107	<0.001	0.047	0.020–0.107	<0.001
**Leukemia-free survival**						
Donor-recipient sex-matched graft						
Female-male	0.774	0.306–1.962	0.590	1.344	0.499–3.568	0.566
Female-female	2.363	1.072–5.209	0.033	3.166	1.414–7.086	0.005
Male-male	1.505	0.708–3.196	0.288	2.426	1.088–5.408	0.030
Male-female		1		1	
Platelet engraftment (yes vs. no)	0.067	0.033–0.133	<0.001	0.054	0.025–0.115	<0.001
**Overall survival**						
Disease status	1.956	0.980–3.905	0.057			
Donor-recipient sex-matched graft						
Female-male	0.492	0.168–1.439	0.195	0.898	0.290–2.780	0.851
Female-female	1.823	0.799–4.158	0.154	2.471	1.067–5.723	0.035
Male-male	1.352	0.629–2.909	0.440	2.290	1.006–5.215	0.048
Male-female		1			1	
Platelet engraftment (yes vs. no)	0.059	0.029–0.119	<0.001	0.050	0.023–0.110	<0.001

*allo-SCT, allogeneic stem cell transplantation; MRD, measurable residual disease; HR, hazard ratio; CI, confidence interval*.

* *All variables were first included in the univariate analysis, including sex, age, donor-recipient sex-matched grafts, donor-recipient relationship, ABO matched graft, pre-transplantation MRD status, disease status and hematopoietic engraftments; only variables with P < 0.1 were included in the Cox proportional hazards model with time-dependent variables*.

### Correlation Between Pre-MRD and Clinical Outcomes After MSDT

**Figure 2:**
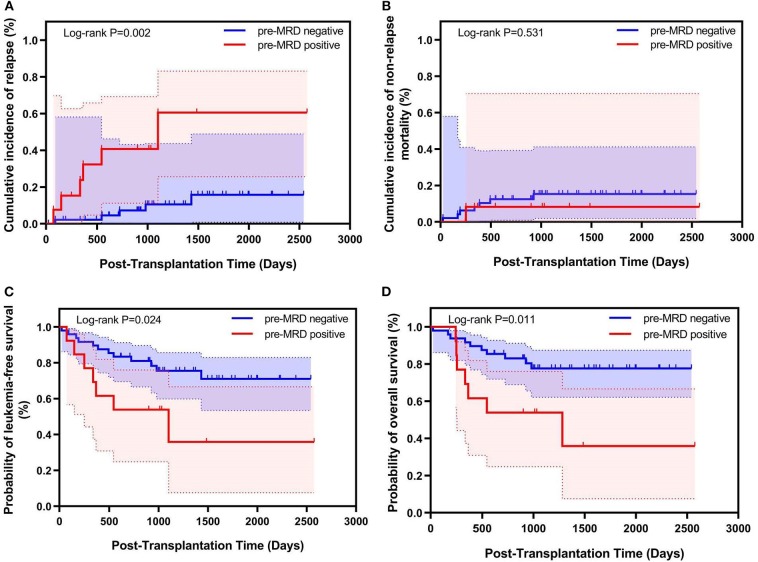
Relationship between pre-transplantation MRD and transplant outcomes for Ph-positive ALL patients who underwent MSDT (*n* = 61). Kaplan–Meier estimates of **(A)** cumulative incidence of relapse mortality, **(B)** cumulative incidence of non-relapse, **(C)** leukemia-free survival, and **(D)** overall survival.

In 61 patients who were treated with MSDT. The cumulative 100-day incidence of grade II-IV acute GVHD (19.1% vs. 23.8%, *P* = 0.760) and 4-year cumulative incidence of chronic GVHD (56.5% vs. 38.3%, *P* = 0.643) were comparable between the pre-MRD negative group and the pre-MRD positive group. Patients with negative pre-MRD experienced a significantly lower 4-year CIR (13.8% vs. 56.4%, *P* < 0.001) as well as higher 4-year LFS (71.0% vs. 35.9%, *P* = 0.024) and OS (77.6% vs. 35.9%, *P* = 0.011). The 4-year NRM was similar in the pre-MRD negative and pre-MRD positive groups (15.2% vs. 7.7%, *P* = 0.654) (Figure 2 and Table 3). Multivariate analysis indicated that positive pre-MRD status was associated with higher CIR (HR, 6.049; *P* = 0.003) and lower LFS (HR, 2.797; *P* = 0.031) and OS (HR, 3.256; *P* = 0.017) (Table 4). In addition, subgroup analysis of Ph-positive patients in CR1 showed that patients with positive pre-MRD had a higher 4-year CIR compared to those with negative pre-MRD in the MSDT subset (33.3% vs. 14.0%, *P* = 0.055) (Table S1). Multivariate analysis showed that positive pre-MRD status was related to higher CIR (HR, 4.006; *P* = 0.058) in cases who receiving MSDT (*n* = 56).

**Table 3:** Transplant outcomes for patients that underwent allogeneic stem cell transplantation (*n* = 202).

		**Neutrophil engraftment**	**Platelet engraftment**	**Grades 2–4 acute GVHD**	**Chronic GVHD**	**Relapse at 4 years**	**NRM at 4 years**	**LFS at 4 years**	**OS at 4 years**
MSDT (*n* = 61)	Pre-MRD neg (Group1, *n* = 48)	100% (95%CI, 100%)	97.9% (95%CI, 93.8–100%)	19.1% (95%CI, 7.9–30.3%)	56.5% (95%CI, 40.4–72.6%)	13.8% (95%CI, 1.6–26.0%)^a^	15.2% (95%CI, 4.6–25.8%)	71.0% (95%CI, 56.3–85.7%)^b^	77.6% (95%CI, 65.3–89.9%)^c^
	Pre–MRD pos (Group2, *n* = 13)	100% (95%CI, 100%)	100% (95%CI, 100%)	23.8% (95%CI, 0.1–47.5%)	38.3% (95%CI, 7.7–68.9%)	56.4% (95%CI, 15.8–97.0%)	7.7% (95%CI, 0–23.0%)	35.9% (95%CI, 2.0–69.8%)	35.9% (95%CI, 2.0–69.8%)
Haplo-HSCT (*n* = 141)	Pre-MRD neg (Group3, *n* = 100)	100% (95%CI, 100%)	90.1% (95%CI, 83.8–96.4%)	22.1% (95%CI, 13.9–30.3%)	44.9% (95%CI, 33.5–56.3%)	10.7% (95%CI, 3.8–17.6%)^a^	16.3% (95%CI, 9.0–23.6%)	72.3% (95%CI, 63.1–81.5%)^d^	75.7% (95%CI, 66.3–85.1%)^e^
	Pre-MRD pos (Group4, *n* = 41)	100% (95%CI, 100%)	94.9% (95%CI, 87.8–100%)	22.0% (95%CI, 9.3–34.7%)	38.5% (95%CI, 21.4–55.6%)	14.8% (95%CI, 3.6–26.0%)^f^	7.3% (95%CI, 0–15.3%)	77.7% (95%CI, 64.8–90.6%)^g^	80.5% (95%CI, 68.3–92.7%)^h^

a *P < 0.001 compared with the Pre-MRDpos MSDT group*.

b *P = 0.024 compared with the Pre-MRDpos MSDT group*.

c *P = 0.011 compared with the Pre-MRDpos MSDT group*.

d *P = 0.043 compared with the Pre-MRDpos MSDT group*.

e *P = 0.020 compared with the Pre-MRDpos MSDT group*.

f *P = 0.021 compared with the Pre-MRDpos MSDT group*.

g *P = 0.036 compared with the Pre-MRDpos MSDT group*.

h *P = 0.027 compared with the Pre-MRDpos MSDT group*.

*MSDT, human leukocyte antigen matched sibling donor transplantation; haplo-HSCT, haploidentical stem cell transplantation; MRD, minimal residual disease; Pre-MRD pos, positive MRD status before transplantation; Pre-MRD neg, negative MRD status before transplantation; GVHD, graft-vs.-host disease; NRM= non-relapse mortality*.

**Table 4:** Multivariate analysis of factors associated with outcomes of patients with Ph positive ALL who underwent MSDT (*n* = 61).

**Covariate**	**Univariate analysis**			**Multivariate analysis**		
	**HR**	**95% CI**	***P*-value**	**HR**	**95% CI**	***P*-value**
**Relapse**						
Disease status (CR > 1 vs. CR1)^*^	5.672	1.455–22.117	0.012			
Pre-transplantation MRD (positive vs. negative)	6.049	1.829–20.007	0.003	6.049	1.829–20.007	0.003
**Leukemia-free survival**						
Pre-transplantation MRD (positive vs. negative)	2.797	1.096–7.140	0.031	2.797	1.096–7.140	0.031
**Overall survival**						
Pre-transplantation MRD (positive vs. negative)	3.256	1.234–8.594	0.017	3.256	1.234–8.594	0.017
Sex (male vs. female)	0.430	0.159–1.164	0.097			

*MSDT, human leukocyte antigen-matched sibling donor transplantation; HR, hazard ratio; CI, confidence interval*.

* *All variables were first included in the univariate analysis, including sex, age, donor-recipient sex-matched grafts, donor-recipient relationship, ABO matched graft, pre-transplantation MRD status, disease status and engraftments; only variables with P < 0.1 were included in the Cox proportional hazards model with time-dependent variables*.

### Correlation Between Pre-MRD and Clinical Outcomes After haplo-SCT

The cumulative 100-day incidence of grade II-IV acute GVHD (22.0% vs. 22.1%, *P* = 0.971) and 4-year cumulative incidence of chronic GVHD (38.5% vs. 44.9%, *P* = 0.687) were comparable. Patients with positive pre-MRD had similar transplant outcomes compared to those without positive pre-MRD (4-year CIR 14.8% vs. 10.7%, *P* = 0.297; 4-year NRM 7.3% vs. 16.3%, *P* = 0.187; 4-year LFS 77.7% vs. 72.3%, *P* = 0.660; 4-year OS 80.5% vs. 75.7%, *P* = 0.804) (Table 3). Multivariate analysis showed that there was no association between pre-MRD positive status and relapse, NRM, LFS, or OS (data not shown). Only disease status (≥CR2 vs. CR1) was associated with higher CIR (HR, 2.604; 95% CI, 1.096–6.183, *P* = 0.030).

### Association of Transplant Modality With Outcomes in Pre-MRD Positive Subgroup

**Figure 3:**
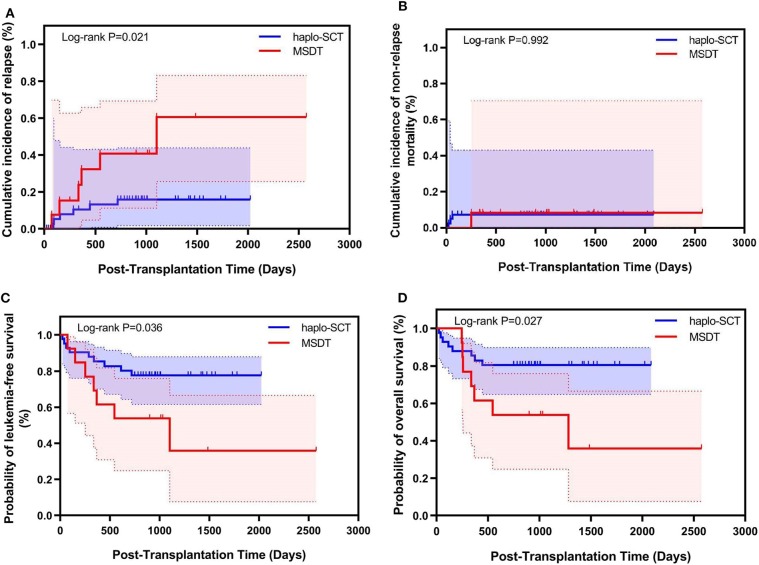
Relationship between transplant modality and transplant outcomes for Ph-positive ALL patients with pre-transplantation MRD who underwent allo-SCT (*n* = 54). Kaplan–Meier estimates of **(A)** cumulative incidence of relapse mortality, **(B)** cumulative incidence of non-relapse, **(C)** leukemia-free survival, and **(D)** overall survival.

Our previous study has shown that compared to MSDT, treating acute myeloid leukemia (AML) patients with positive pre-MRD with haplo-SCT could achieve lower CIR (31). In this study, fifty-four patients had positive pre-MRD, the median level of MRD was 0.03% (0.001–2.210%). There were no difference in the level of pre-MRD between patients who underwent haplo-SCT and those received MSDT (Table 1). However, compared with patients in the haplo-SCT group, more cases in MSDT group received post-transplantation TKIs other than imatinib (*P* = 0.033). In comparison to those with positive pre-MRD undergoing MSDT, patients who were treated with haplo-SCT had a lower 4-year CIR (14.8% vs. 56.4%, *P* = 0.021) and higher 4-year LFS (77.7% vs. 35.9%, *P* = 0.036), OS (80.5% vs. 35.9%, *P* = 0.027). The 4-year NRM was comparable in the MSDT and haplo-SCT groups (7.3% vs. 7.7%, *P* = 0.992) (Figure 3). Multivariate analysis revealed that haplo-SCT was associated with lower CIR (HR, 0.288; *P* = 0.031) and high probability of LFS (HR, 0.283; *P* = 0.019) and OS (HR, 0.252; *P* = 0.013) (Table 5). In Ph-positive patients in CR1 with positive pre-MRD, cases underwent haplo-SCT had a lower 4-year CIR compared to those cases underwent MSDT (9.0% vs. 33.3%, *P* = 0.057). Multivariate analysis showed that haplo-SCT was related to lower CIR (HR, 0.235; *P* = 0.077) (Tables S1, S2). We did not find differences in kinetics of the BCR/ABL levels before day 180 after transplantation between patients with positive pre-MRD who underwent haplo-SCT and those who received MDST. This could be related to the results that, in the current study, 27 patients relapsed, 21 of them relapsed after 180 days post transplantation (Table S3).

**Table 5:** Multivariate analysis of factors associated with outcomes of Ph positive ALL patients with positive pre-transplantation MRD who underwent allo-SCT (*n* = 54).

**Covariate**	**Univariate analysis**			**Multivariate analysis**		
	**HR**	**95% CI**	***P*-value**	**HR**	**95% CI**	***P*-value**
**Relapse**						
Transplant modality (haplo-SCT vs. MSDT)^*^	0.288	0.093–0.895	0.031	0.288	0.093–0.895	0.031
Disease status (CR > 1 vs. CR1)	2.304	0.989–5.366	0.053			
**Transplant-related mortality**						
Platelet engraftment (yes vs. no)	0.072	0.007–0.707	0.024	0.072	0.007–0.707	0.024
**Leukemia-free survival**						
Transplant modality (haplo-SCT vs. MSDT)	0.363	0.135–0.977	0.045	0.283	0.099–0.810	0.019
Platelet engraftment (yes vs. no)	0.090	0.019–0.428	0.002	0.056	0.011–0.293	0.001
**Overall survival**						
Transplant modality (haplo-SCT vs. MSDT)	0.334	0.121–0.924	0.035	0.252	0.084–0.752	0.013
Platelet engraftment (yes vs. no)	0.090	0.019–0.425	0.002	0.052	0.010–0.276	0.001

*MSDT, human leukocyte antigen-matched sibling donor transplantation; halo-SCT, haploidentical stem cell transplantation; HR, hazard ratio; CI, confidence interval*.

* *All variables were first included in the univariate analysis, including sex, age, donor-recipient sex-matched grafts, donor-recipient relationship, ABO matched graft, transplantation modality, disease status and engraftments; only variables with P < 0.1 were included in the Cox proportional hazards model with time-dependent variable*.

### Correlation Between Pre-MRD and Clinical Outcomes in a Propensity Score Matched Analysis

Sixty-one patients who underwent MSDT and 61 patients who received haplo-SCT were enrolled in the propensity score matched analysis. For patients who were treated with MSDT, cases with positive pre-MRD had a significantly higher 4-year CIR (56.4% vs. 13.8%, *P* = 0.008) and lower 4-year LFS (35.9% vs. 71.0%, *P* = 0.024) and OS (35.9% vs. 77.6%, *P* = 0.011) compared to those with negative pre-MRD. However, in haplo-SCT subgroup, patients with positive pre-MRD had similar transplant outcomes compared to those with negative pre-MRD (CIR 15.4% vs. 13.5%, *P* = 0.683; NRM 15.4% vs. 18.8%, *P* = 0.843; OS 84.6% vs. 69.9%, *P* = 0.468; LFS 69.2% vs. 67.8%, *P* = 0.880). Univariate analysis showed that, in the pre-MRD positive subgroup, patients who were treated with haplo-SCT had a lower 4-year CIR than that of cases received MSDT (15.4% vs. 56.4%, *P* = 0.002) (Table S4). Multivariate analysis was not performed considering there were 26 patients with positive pre-MRD.

## Discussion

In agreement with previous reports (20, 26), the results of our study indicated that positive pre-MRD, detected by MFC, was associated with higher CIR in Ph-positive ALL patients who underwent allo-SCT. In the MSDT group, positive pre-MRD was not only associated with higher CIR but also related to lower survival rates. Surprisingly, we observed no negative effects of positive pre-MRD on outcomes in haplo-SCT treatment cases. Subgroup analysis of pre-MRD positive cases showed that, compared to MSDT, haplo-SCT was associated with lower CIR and superior survival. Overall, our data not only showed that there are different effects of positive pre-MRD on outcomes according to transplant modality but also suggested that haplo-SCT might have stronger graft-vs.-leukemia (GVL) effects compared to MSDT, as previously reported by others (47) and us (48, 49), although controversy remains (50).

A number of previous studies confirm a negative effect of positive pre-MRD in patients undergoing allo-SCT (9–27). In a recent meta-analysis of 21 studies, Shen et al. (51) found that in HLA-matched allo-SCT settings, positive pre-MRD was related to higher CIR (HR, 3.26; *P* < 0.05) as well as lower relapse-free survival (HR, 2.53; *P* < 0.05), LFS (HR, 4.77; *P* < 0.05), and OS (HR, 1.98; *P* < 0.05). In the current study, we found that Ph-positive ALL cases with positive pre-MRD in the MSDT group experienced higher CIR and lower OS and LFS. Therefore, the results reported by others (15, 27) and us suggest that in HLA-matched allograft modalities, positive pre-MRD is related to inferior survival regardless of the condition regimen, GVHD prophylaxis, and sources of stem cells, such as sibling donors, unrelated donors, and cord blood. Fortunately, the efficacies of blinatumomab and chimeric antigen receptor T-cells (CAR-T) in relapsed, refractory, or MRD-positive ALL have been confirmed by several studies (52, 53), which provide novel strategies for resolving a positive pre-MRD status to a negative one to improve transplant outcomes.

Previous studies by others (47, 54) and us (48, 49, 55) showed that, given AML patients with positive pre-MRD or subjects with Hodgkin's disease who relapsed after autologous SCT, patients receiving haplo-SCT experienced lower CIR and superior survival compared to patients receiving MDST, although controversy remains (50). Interestingly, we found that treatment based on haplo-SCT could overcome the negative effects of a positive pre-MRD diagnosis on relapse in patients with Ph-positive ALL and lead to better survival (Figure 2). This result was not replicated in patients treated instead with MDST, as found via subgroup analysis in positive pre-MRD Ph-positive ALL cases. The results of our study add further evidence to previous studies suggesting that, compared to MDST, haplo-SCT might have stronger GVL effects. Several reasons may account for the stronger GVL effects of haplo-SCT: First, Dabas et al. (56) demonstrated that anti-thymocyte globulin (ATG) at clinically relevant concentrations kills leukemic blasts. In this study, ATG was only used in the haplo-SCT setting. Second, the stronger GVL effects of haplo-SCT might be ascribed to the large number of alloreactive T-cell targets encoded by the fully mismatched haplotype and/or HLA disparity (54). Third, alloreactive natural killer (NK) cells may also play an important role in anti-leukemia activity in haplo-SCT settings (47).

Presently, some researchers demonstrated that 3-year OS was 83% in patients who did not undergo SCT in first remission (ASH 2019), however, the current recommendation is the pursue allo-SCT for Ph^+^ ALL (57–59). A consensus of North American experts also indicates that allo-SCT is an alternative method either for Ph+ ALL with negative pre-transplant MRD or for cases with positive pre-transplant MRD (7). Of course, the administration of TKIs in our study may contribute to improved survival according to previous study (60).

There are still limitations in our study. First, this study is a retrospective study and was conducted at a single center. Second, the haplo-SCT protocols are based on the utility of granulocyte colony-stimulating factor and ATG. Third, the detection of post-transplantation MRD is based on RT-PCR or MFC only. It would be more precise to evaluate the pre-MRD level by combining MFC with the utility of RQ-PCR. A multicenter prospective study is needed to confirm our findings in haplo-SCT modalities, including haplo-SCT with post-cyclophosphamide.

In conclusion, our results indicate that the effects of positive pre-MRD on outcomes are different according to transplant modality. For Ph-positive ALL patients with positive pre-MRD, haplo-SCT was related to lower incidences of relapse and a higher probability of survival. This suggests that haplo-SCT has a stronger GVL effect based on this study and previous reports (48, 49). This study provides novel evidence supporting the claim that, for Ph-positive ALL patients with positive pre-MRD, haplo-SCT is a better option than MSDT, especially for patients without HLA-identical sibling donors.

## Data Availability Statement

All datasets generated for this study are included in the article/Supplementary Material.

## Ethics Statement

The studies involving human participants were reviewed and approved by Institutional Review Board of Peking University. Written informed consent to participate in this study was provided by the participants' legal guardian/next of kin. Written informed consent was obtained from the individual(s), and minor(s)' legal guardian/next of kin, for the publication of any potentially identifiable images or data included in this article.

## Author Contributions

Y-JC designed the study. S-QL, Q-ZF, and Y-JC collected the data. S-QL, Q-ZF, Y-JC, and X-JH analyzed the data and drafted the manuscript. All authors contributed to the data interpretation, manuscript preparation, and approval of the final version.

### Conflict of Interest

The authors declare that the research was conducted in the absence of any commercial or financial relationships that could be construed as a potential conflict of interest.
