# Supplementary material for: Different Effects of Pre-transplantation Measurable Residual Disease on Outcomes According to Transplant Modality in Patients With Philadelphia Chromosome Positive ALL
Source: Front Oncol. 2020 Mar 17;10:320. doi: 10.3389/fonc.2020.00320 (PMC7089930; doi:10.3389/fonc.2020.00320)
Supplement: Supplementary file 1 [file Table_1.DOC]

**Table S1. Transplant outcomes for patients in CR1 that underwent allogeneic stem cell transplantation (n=188)**

|  |  | **Neutrophil**  **engraftment** | **Platelet**  **engraftment** | **Grades 2–4**  **acute GVHD** | **Chronic GVHD** | **Relapse**  **at 4 years** | **TRM**  **at 4 years** | **LFS**  **at 4 years** | **OS**  **at 4 years** |
| --- | --- | --- | --- | --- | --- | --- | --- | --- | --- |
| **MSDT**  **(n=56)** | Pre-MRD neg  (Group1,n=47) | 100% (95%CI,  100%) | 100% (95%CI,  100%) | 20.3% (95%CI,  8.3%-32.3%) | 55.5% (95%CI,  39.2%-71.8%) | 14.0% (95%CI,  1.7%-26.3%) ^a^ | 15.5% (95%CI,  4.7%-26.3%) | 70.5% (95%CI,  55.6%-85.4%) | 77.1% (95%CI,  64.4%-89.8%) |
|  | Pre-MRD pos  (Group2, n=9) | 100% (95%CI,  100%) | 100% (95%CI,  100%) | 13.8% (95%CI,  0.0%-42.8%) | 30.0% (95%CI,  0.0%-65.7%) | 33.3% (95%CI,  0.0%-66.6%) | 11.1% (95%CI,  0.0%-66.6%) | 55.6% (95%CI,  23.1%-88.1%) | 55.6% (95%CI,  23.1%-88.1%) |
| **Haplo-HSCT**  **(n=132)** | Pre-MRD neg  (Group3, n=98) | 100% (95%CI,  100%) | 97.8% (95%CI,  93.9%-100%) | 28.3% (95%CI,  17.9%-38.7%) | 46.0% (95%CI,  34.4%-57.6%) | 10.9% (95%CI,  4.0%-17.8%) ^b^ | 15.6% (95%CI,  8.3%-22.9%) | 72.8% (95%CI,  63.6%-82.0%) | 76.2% (95%CI,  66.6%-85.8%) |
|  | Pre-MRD pos  (Group4, n=34) | 100% (95%CI,  100%) | 96.9% (95%CI,  90.8%-100%) | 17.2% (95%CI,  3.3%-31.1%) | 42.6% (95%CI,  24.0%-61.2%) | 9.0% (95%CI,  0.0%-19.0%) ^c^ | 8.8% (95%CI,  0%-18.6%) | 79.4% (95%CI,  65.9%-92.9%) | 85.3% (95%CI,  73.3%-97.3%) ^d^ |

**a**  P = 0.055 compared with the Pre-MRDpos MSDT group

**b** P = 0.018 compared with the Pre-MRDpos MSDT group

**c**  P = 0.057 compared with the Pre-MRDpos MSDT group

**d** P = 0.064 compared with the Pre-MRDpos MSDT group

**Abbreviation**s: MSDT= human leukocyte antigen matched sibling donor transplantation; haplo-HSCT= haploidentical stem cell transplantation; MRD= minimal residual disease; Pre-MRD pos= positive MRD status before transplantation; Pre-MRD neg= negative MRD status before transplantation; GVHD= graft-versus-host disease; TRM= treatment-related mortality
