# Supplementary material for: Different Effects of Pre-transplantation Measurable Residual Disease on Outcomes According to Transplant Modality in Patients With Philadelphia Chromosome Positive ALL
Source: Front Oncol. 2020 Mar 17;10:320. doi: 10.3389/fonc.2020.00320 (PMC7089930; doi:10.3389/fonc.2020.00320)
Supplement: Supplementary file 2 [file Table_2.DOC]

**Table S2. Multivariate analysis of factors associated with outcomes of Ph positive ALL patients in CR1 with positive pre-transplantation MRD who underwent allo-SCT (n=43)**

| **Covariate** | **Univariate analysis** | | |  | **Multivariate analysis** | | |
| --- | --- | --- | --- | --- | --- | --- | --- |
|  | HR | 95% CI | *P*-value |  | HR | 95% CI | *P*-value |
| **Relapse** |  |  |  |  |  |  |  |
| Transplant mortality (haplo-SCT vs. MSDT) | 0.235 | 0.047-1.169 | 0.077 |  | 0.235 | 0.047-1.169 | 0.077 |
| **Transplant-related mortality** |  |  |  |  |  |  |  |
| Platelet engraftment (yes vs. no) | 0.090 | 0.009-0.888 | 0.039 |  | 0.090 | 0.009-0.888 | 0.039 |
| **Leukemia-free survival** |  |  |  |  |  |  |  |
| Platelet engraftment (yes vs. no) | 0.072 | 0.014-0.374 | 0.002 |  | 0.072 | 0.014-0.374 | 0.002 |
| **Overall survival** |  |  |  |  |  |  |  |
| Transplant mortality (haplo-SCT vs. MSDT) | 0.308 | 0.082-1.150 | 0.080 |  | 0.184 | 0.041-0.827 | 0.027 |
| Platelet engraftment (yes vs. no) | 0.058 | 0.010-0.318 | 0.001 |  | 0.029 | 0.004-0.203 | <0.001 |

**Abbreviations:** MSDT=human leukocyte antigen-matched sibling donor transplantation; halo-SCT= haploidentical stem cell transplantation; HR=hazard ratio; CI=confidence interval

* All variables were first included in the univariate analysis; only variables with *P* < 0.1 were included in the Cox proportional hazards model with time-dependent variables.
