# Supplementary material for: Different Effects of Pre-transplantation Measurable Residual Disease on Outcomes According to Transplant Modality in Patients With Philadelphia Chromosome Positive ALL
Source: Front Oncol. 2020 Mar 17;10:320. doi: 10.3389/fonc.2020.00320 (PMC7089930; doi:10.3389/fonc.2020.00320)
Supplement: Supplementary file 3 [file Table_3.DOC]

**Table S3**. **The kinetics of the value of MRD for patients with positive pre-MRD (n=54)**

| Post-Transplantation Time | MSDT | Haplo-SCT | P |
| --- | --- | --- | --- |
| +30Days (n=52) | 0.000 (0.000-0.480) | 0.000 (0.000-0.460) | 0.493 |
| +60Days (n=50) | 0.000 (0.000-2.300) | 0.000 (0.000-1.400) | 0.360 |
| +90Days (n=45) | 0.000 (0.000-1.000) | 0.000 (0.000-7.300) | 0.822 |
| +120Days (n=44) | 0.000 (0.000-0.370) | 0.000 (0.000-3.700) | 0.378 |
| +180Days (n=31) | 0.006 (0.000-3.900) | 0.006 (0.000-8.800) | 0.069 |

Abbreviations: MSDT=HLA-matched sibling donor transplantation, Haplo-SCT= unmanipulated haploidentical stem cell transplantation, MRD= minimal residual disease
