# Supplementary material for: Different Effects of Pre-transplantation Measurable Residual Disease on Outcomes According to Transplant Modality in Patients With Philadelphia Chromosome Positive ALL
Source: Front Oncol. 2020 Mar 17;10:320. doi: 10.3389/fonc.2020.00320 (PMC7089930; doi:10.3389/fonc.2020.00320)
Supplement: Supplementary file 4 [file Table_4.DOC]

**Table S4. Transplant outcomes for patients that underwent allogeneic stem cell transplantation in propensity score matched group (n=122).**

|  | |  | **Neutrophil**  **engraftment** | **Platelet**  **engraftment** | **Grades 2–4**  **acute GVHD** | **Chronic GVHD** | **Relapse**  **at 4 years** | **NRM**  **at 4 years** | **LFS**  **at 4 years** | **OS**  **at 4 years** |
| --- | --- | --- | --- | --- | --- | --- | --- | --- | --- | --- |
| **MSDT**  **(n=61)** | Pre-MRD neg  (Group1, n=48) | | 100% (95%CI,  100%) | 100% (95%CI,  100%) | 20.3% (95%CI,  8.5%-32.1%) | 56.5% (95%CI,  40.4%-72.6%) | 13.8% (95%CI,  1.6%-26.0%)^a^ | 15.2% (95%CI,  4.6%-25.8%) | 71.0% (95%CI,  56.3%-85.7%) ^b^ | 77.6% (95%CI,  65.3%-89.9%) ^c^ |
|  | Pre-MRD pos  (Group2, n=13) | | 100% (95%CI,  100%) | 100% (95%CI,  100%) | 23.8% (95%CI,  0.1%-47.5%) | 38.3% (95%CI,  7.7%-68.9%) | 56.4% (95%CI,  15.8%-797.0%) | 7.7% (95%CI,  0%-23.0%) | 35.9% (95%CI,  2.0%-69.8%) | 35.9% (95%CI,  2.0%-69.8%) |
| **Haplo-HSCT**  **(n=61)** | Pre-MRD neg  (Group3, n=48) | | 100% (95%CI,  100%) | 96.1% (95%CI,  89.0%-100%) | 26.2% (95%CI,  12.7%-39.7%) | 39.5% (95%CI,  24.2%-54.8%) | 13.5% (95%CI,  3.3%-23.7%) ^d^ | 18.8% (95%CI,  7.6%-30.0%) | 67.8% (95%CI,  54.3%-81.3%) | 69.9% (95%CI,  55.2%-84.6%) |
|  | Pre-MRD pos  (Group4, n=13) | | 100% (95%CI,  100%) | 100% (95%CI,  100%) | 16.7% (95%CI,  0%-37.9%) | 37.7% (95%CI,  8.3%-67.1%) | 15.4% (95%CI,  0%-36.0%) ^e^ | 15.4% (95%CI,  0%-35.8%) | 69.2% (95%CI,  44.1%-94.3%) | 84.6% (95%CI,  65.0%-100%) |

**a**  P = 0.008 compared with the Pre-MRDneg MSDT group

**b** P = 0.024 compared with the Pre-MRDneg MSDT group

**c**  P = 0.011 compared with the Pre-MRDneg MSDT group

**d** P = 0.003 compared with the Pre-MRDpos MSDT group

**e**  P = 0.002 compared with the Pre-MRDpos MSDT group

**Abbreviations**: MSDT= human leukocyte antigen matched sibling donor transplantation; haplo-HSCT= haploidentical stem cell transplantation; MRD= minimal residual disease; Pre-MRD pos= positive MRD status before transplantation; Pre-MRD neg= negative MRD status before transplantation; GVHD= graft-versus-host disease; NRM= non-relapse mortality
